# Skew Constacyclic Codes over a Non-Chain Ring

**DOI:** 10.3390/e25030525

**Published:** 2023-03-17

**Authors:** Mehmet Emin Köroğlu, Mustafa Sarı

**Affiliations:** Department of Mathematics, Faculty of Art and Sciences, Yildiz Technical University, Istanbul 34220, Turkey

**Keywords:** non-chain ring, skew constacyclic codes, LCD codes, entanglement-assisted quantum codes

## Abstract

In this paper, we investigate the algebraic structure of the non-local ring $R_{q}=F_{q}\left[v\right]/\langle v^{2}+1\rangle$ and identify the automorphisms of this ring to study the algebraic structure of the skew constacyclic codes and their duals over this ring. Furthermore, we give a necessary and sufficient condition for the skew constacyclic codes over $R_{q}$ to be linear complementary dual (LCD). We present some examples of Euclidean LCD codes over $R_{q}$ and tabulate the parameters of Euclidean LCD codes over finite fields as the Φ-images of these codes over $R_{q}$, which are almost maximum distance separable (MDS) and near MDS. Eventually, by making use of Hermitian linear complementary duals of skew constacyclic codes over $R_{q}$ and the map Φ, we give a class of entanglement-assisted quantum error correcting codes (EAQECCs) with maximal entanglement and tabulate parameters of some EAQECCs with maximal entanglement over finite fields.

## 1. Introduction

In recent decades, codes over finite commutative chain rings have been studied considerably (see Refs. [1,2,3,4,5,6,7]). In the last few years, some specific non-chain rings have been used as an alphabet for codes (see Refs. [8,9,10,11,12]). Constacyclic codes form an important class of linear codes and have practical applications to other disciplines including classical and quantum communication systems as they can be encoded with shift registers because of their algebraic structures. Since the factorization of the polynomials over non-commutative structures is not unique, they are potentially more convenient for obtaining good code parameters than commutative structures. This fact makes the study of skew polynomial rings more attractive. Over standard polynomial rings the algebraic structure of λ-constacyclic codes of length *n* is totally determined by the polynomial divisors of the binomial $x^{n}-\lambda .$ In [13], Boucher, Solé and Ulmer used skew polynomials to determine the algebraic structure of constacyclic codes under a skew constacyclic shift. Afterwards, in [14,15], Boucher and Ulmer explored more properties and good examples of such codes.

For the first time, linear complementary dual (LCD) codes over finite fields were introduced by Massey in [16]. In recent years, many researches have been conducted to obtain conditions for certain families of linear codes to be LCD. For a cyclic code, the necessary and sufficient condition to be an LCD code was derived by Yang and Massey in [17]. Zhu et al. in [18] and Koroglu and Sarı in [19] constructed some classes of maximum distance separable (MDS) LCD codes from negacyclic codes. Esmaeili and Yari studied on quasi-cyclic linear complementary dual codes [20]. For a list of papers on LCD codes from other families of linear codes see Refs. [21,22,23,24,25,26,27].

Recently entanglement-assisted quantum error-correcting codes (EAQECCs) have been studied vigorously by researchers, see Refs. [28,29,30,31,32,33,34,35,36,37,38,39,40,41,42,43,44,45]. Especially, the construction of EAQECCs from LCD codes has been the main focus of attention since the number of pairs of maximally entangled states of an EAQECC derived from an LCD code of length *n* and dimension *k* is n−k, which give us the occasion to construct EAQECCs with maximal entanglement [33,38,44]. In [44], Qian and Zhang showed that a λ-constacyclic code over $F_{q^{2}}$ is a Hermitian LCD code if the multiplicative order of λ does not divide q+1, and by the help of this fact, they obtained new entanglement-assisted quantum maximum distance separable codes of length q+1 from MDS Hermitian LCD codes. In [46], Sarı and Koroglu expanded the range of parameters by considering the defining sets given in [44] with a different approach.

The rest of the paper is organized as follows. In Section 2, we recall some basic notations and results that are needed in the remaining part of the study. In Section 3, we remind the algebraic structure of the ring $R_{q}$ and then give a decomposition of it. In the same section, we determine automorphism group of the ring and define a Gray type map over it. Further, we recall some results regarding to the algebraic structure of the linear codes over the ring $R_{q}.$ In Section 4, we introduce basics of the skew constacyclic codes over finite fields. In Section 5, we define LCD codes over $R_{q}$ and give a characterization for skew constacyclic codes over $R_{q}$ to be Euclidean LCD and Hermitian LCD. We also tabulate some parameters of almost maximum distance separable (MDS) and near MDS LCD codes over $F_{169}$. In Section 6, we apply the skew constacyclic Hermitian LCD codes over $R_{q}$ to obtain EAQECCs and give some parameters of EAQECCs over $F_{5}$.

## 2. Preliminaries

In this section, we will fix some notations for the sequel of the paper and recall some basic notations and results that are needed in the rest of the study. Throughout this work, we will use the following notation unless otherwise noted.

- $q=p^{k}$ is a prime power and for positive integers *a* and *b*, where $p=a^{2}+b^{2}$.
- $F_{q}$ is the finite field of *q* elements.
- $F_{q}^{*}=F_{q}-\left\{0\right\}$.
- $R_{q}=F_{q}\left[v\right]/\langle v^{2}+1\rangle$ such that $v^{2}\equiv -1(mod$q).
- $U(R_{q})$ is the unit group of $R_{q}$.
- $Aut\left(R_{q}\right)$ is the automorphism group of $R_{q}$.

A linear code of length *n* and dimension *k* over $F_{q}$ is a vector subspace of the vector space $F_{q}^{n}.$ An element of a linear code is termed as a codeword. The minimum Hamming distance *d* of a linear code C is the minimum Hamming weight $w_{H}\left(C\right)$ of C, where $w_{H}\left(C\right)=\min\left\{w_{H}\left(c\right):0\neq c\in C\right\}$ and $w_{H}\left(c\right)=\left|\left\{i:c_{i}\neq 0,\;i\in \left\{0,1,\ldots ,n-1\right\}\right\}\right|.$ A linear code C over $F_{q}$ of length *n*, dimension *k* and minimum distance *d* is denoted by the triple ${\left[n,k,d\right]}_{q}$ and this code corrects up to $\left\lfloor \frac{d-1}{2}\right\rfloor$ errors. For an ${\left[n,k,d\right]}_{q}$ linear code *C*, if d=n−k+1, then it is called a maximum distance separable (MDS) code. We say that it is an almost maximum distance separable (MDS) code if d=n−k, and it is a near MDS code if d=n−k−1. The (Euclidean) dual $C^{⊥}$ of a linear code C over $F_{q}$ of length *n* is the set

$$C^{⊥}=\left\{y\in F_{q}^{n}:\sum_{i=0}^{n-1}x_{i}y_{i}=0,\;\forall x\in C\right\}.$$

The (Hermitian) dual $C^{{⊥}_{h}}$ of a linear code C over $F_{q^{2}}$ of length *n* is the set

$$C^{{⊥}_{h}}=\left\{y\in F_{q^{2}}^{n}:{\langle x,y\rangle }_{h}=0,\;\forall x\in C\right\},$$

where ${\langle x,y\rangle }_{h}=\sum_{i=0}^{n-1}x_{i}y_{i}^{q}.$ Note that the dual of a linear code is also linear. For a linear code C over $F_{q^{2}}$, a Hermitian parity check matrix *H* is a matrix whose rows form a basis of $C^{{⊥}_{h}}.$ Conjugate transpose of an m×n matrix $H=\left(h_{ij}\right)$ with entries in $F_{q^{2}}$ is denoted by $H^{†}$ and is an n×m matrix such that $H^{†}=\left(h_{ji}^{q}\right).$

Let $\lambda \in F_{q}$ be a nonzero element. Then a λ-constacyclic code over the finite field $F_{q}$ of length *n* is a linear code C satisfying that $\left(\lambda c_{n-1},c_{0},\ldots ,c_{n-2}\right)\in C$ for each codeword $c=\left(c_{0},\ldots ,c_{n-1}\right)\in C.$ By mapping a codeword $c=\left(c_{0},\ldots ,c_{n-1}\right)$ to a polynomial $c_{0}+c_{1}x+\cdots +c_{n-1}x^{n-1},$ one gets that a λ-constacyclic code over $F_{q}$ of length *n* corresponds to a principal ideal $C=\langle g\left(x\right)\rangle$ in the quotient ring $F_{q}\left[x\right]/\langle x^{n}-\lambda \rangle$. Note that a constacyclic code $C=\langle g\left(x\right)\rangle$ of length *n* is of n−k dimension, where $k=deg(g\left(x\right)).$ For λ=1 the code C is called a cyclic code and for λ=−1 the code C is called a negacyclic.

## 3. Structure of the Ring $R_{q}$ and Linear Codes over $R_{q}$

In this section, we remind algebraic structure of the ring $R_{q}$ and we give a decomposition of it. We determine automorphism group of the ring and define a Gray type map over it. Finally, we recall structure of the linear codes over the ring $R_{q}.$

An automorphism of the finite field $F_{q}$ is a bijection from the field onto itself. The distinct automorphisms of $F_{q}$ over $F_{p}$ are exactly the mappings $\theta_{0},\theta_{1},\ldots ,\theta_{k-1}$, defined by $\theta_{j}\left(\beta \right)=\beta^{p^{j}}$ for $\beta \in F_{q}$ and 0⩽j⩽k−1.

**Figure 1:**
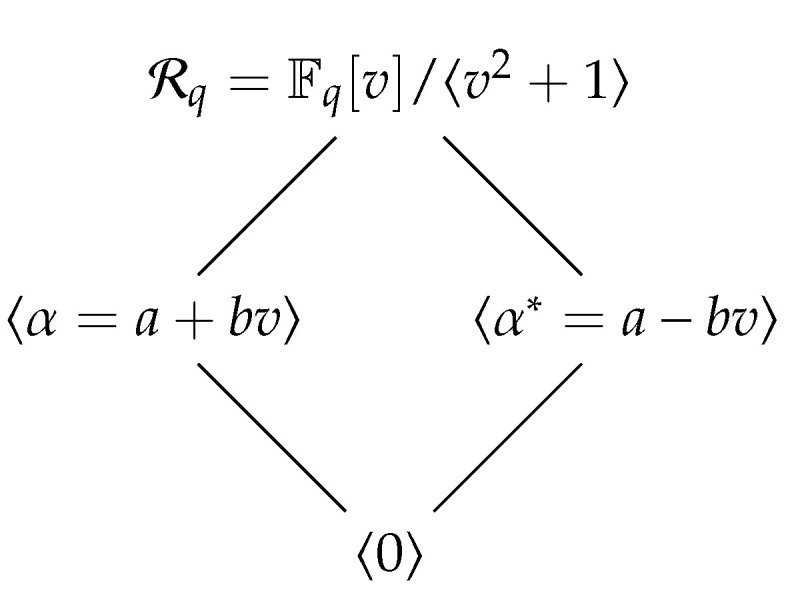
The ideal lattice of the ring $R_{q}=F_{q}\left[v\right]/\langle v^{2}+1\rangle$.

The ring $R_{q}=F_{q}\left[v\right]/\langle v^{2}+1\rangle$ such that $v^{2}\equiv -1\left(modq\right)$ is a non-chain principal ideal ring with two maximal ideals 〈α〉 and $\langle \alpha^{*}\rangle ,$ where α=a+bv is an element of $R_{q}$ and $\alpha^{*}=a-bv$, which is called as the conjugate of the element α. The ideal lattice of $R_{q}$ is given in Figure 1.

An element $\pi \in R_{q}$ is called an idempotent if $\pi^{2}=\pi$ and two idempotents $\pi_{1},$$\pi_{2}$ are said to be orthogonal if $\pi_{1}\pi_{2}=0.$ An idempotent of $R_{q}$ is said to be primitive if it is non-zero and it cannot be written as sum of orthogonal idempotents. A collection $\left\{\pi_{0},\pi_{1},\ldots ,\pi_{s-1}\right\}$ of idempotents of $R_{q}$ is complete if $\pi_{0}+\pi_{1}+\ldots +\pi_{s-1}=1.$ Any complete collection of idempotents in $R_{q}$ is a basis of the $F_{q}$-vector space $R_{q}.$ Hence, any element $r\in R_{q}$ can be uniquely represented as $r=r_{0}\pi_{0}+r_{1}\pi_{1}+\ldots +r_{s-1}\pi_{s-1},$ where $r_{i}\in R_{q}.$ For more details readers may consult reference [11].

Let $\pi_{0}=\frac{1}{2a}\alpha$ and $\pi_{1}=\frac{1}{2a}\alpha^{*}$ be two elements in $R_{q}.$ It is easy to see that the set $\left\{\pi_{0},\pi_{1}\right\}$ is a complete set of idempotents in $R_{q}.$ Therefore, any element $r\in R_{q}$ can be uniquely represented as $r=r_{0}\pi_{0}+r_{1}\pi_{1},$ where $r_{0},r_{1}\in F_{q}.$ From the Figure 1, we can easily see that an element $x\pi_{0}+y\pi_{1}\in R_{q}$ is a unit if and only if both *x* and *y* are nonzero. Then the unit group of $R_{q}$ is described as

$$U\left(R_{q}\right)=\left\{\left.x\pi_{0}+y\pi_{1}\right|x,\;y\in F_{q}\;\mathrm{such}\;\mathrm{that}\;x\neq 0\;\mathrm{and}\;y\neq 0\right\}.$$

Because of the choice of *x* and y, the number of unit elements of $R_{q},$ i.e., the cardinality of the set $U\left(R_{q}\right),$$\left|U\left(R_{q}\right)\right|$ is equal to $\left(q-1\right)\left(q-1\right).$

**Theorem** **1.** *Let θ be an automorphism of $F_{q}$ and σ be a permutation of the set $\left\{0,1\right\}.$ Then the map*

$$\begin{array}{ccc} \Theta_{\theta ,\sigma } & : & R_{q}⟶R_{q}\\ \Theta_{\theta ,\sigma }\left(r_{0}\pi_{0}+r_{1}\pi_{1}\right) & \mapsto & \theta \left(r_{0}\right)\pi_{\sigma \left(0\right)}+\theta \left(r_{1}\right)\pi_{\sigma \left(1\right)} \end{array}$$

 *is an automorphism of the ring* $R_{q}.$ *Further, the cardinality* $\left|Aut\left(R_{q}\right)\right|$ *of the automorphism group of* $R_{q}$

$$Aut\left(R_{q}\right)=\left\{\left.\Theta_{\theta ,\sigma }\right|\theta \in Aut\left(F_{q}\right)\;\mathrm{and}\;\sigma \in S_{2}\right\},$$

 *where* $S_{2}$ *is the permutation group of the set* $\left\{0,1\right\},$ *is* 2k.

**Proof of Theorem 1.**  It is easy to check that $\Theta_{\theta ,\sigma }$ is an automorphism of the ring $R_{q}.$ Hence,

$$\left\{\left.\Theta_{\theta ,\sigma }\right|\theta \in Aut\left(F_{q}\right)\;\mathrm{and}\;\sigma \in S_{2}\right\}\subset Aut\left(R_{q}\right).$$

On the other hand, if $\Theta \in Aut\left(R_{q}\right)$, then the restriction of Θ over $F_{q}$ is θ. Thus, for any $r=r_{0}\pi_{0}+r_{1}\pi_{1}\in R_{q},$ we have $\Theta \left(r\right)=\theta \left(r_{0}\right)\Theta \left(\pi_{0}\right)+\theta \left(r_{1}\right)\Theta \left(\pi_{1}\right).$ Now the set $\left\{\Theta \left(\pi_{0}\right),\Theta \left(\pi_{1}\right)\right\}$ is another complete set of primitive pairwise orthogonal idempotents in $R_{q}.$ By the idempotent decomposition of the ring $R_{q}=\pi_{0}R_{q}⊕\pi_{1}R_{q},$ it follows that there exists a permutation of the set $\left\{0,1\right\}$ such that $\Theta \left(\pi_{i}\right)=\pi_{\sigma \left(i\right)}.$ Therefore, $\Theta \left(r\right)=\theta \left(r_{0}\right)\pi_{\sigma \left(0\right)}+\theta \left(r_{1}\right)\pi_{\sigma \left(1\right)}$ and $Aut\left(R_{q}\right)=\left\{\left.\Theta_{\theta ,\sigma }\right|\theta \in Aut\left(F_{q}\right)\;\mathrm{and}\;\sigma \in S_{2}\right\}.$ Eventually, $\Theta_{\theta ,\sigma }\circ \Theta_{\theta^{\prime },\sigma^{\prime }}=\Theta_{\theta \circ \theta^{\prime },\sigma \circ \sigma^{\prime }}$ and hence $\left|Aut\left(R_{q}\right)\right|=2k.$ □

**Example** **1.** *Let a=2, b=1, p=5 and q=25. Then, $\left\{\pi_{0}=3+4v,\;\pi_{1}=3+v\right\}$ is a complete set of idempotents of the ring $R_{25}$. The maximal ideals of $R_{25}$ are $\langle \pi_{0}\rangle =\left\{k\left(3+4v\right):k\in F_{5}\right\}$ and $\langle \pi_{1}\rangle =\left\{l\left(3+v\right):l\in F_{5}\right\}$. Morevoer, $U\left(R_{25}\right)=16$ and $\left|Aut\left(R_{q}\right)\right|=4$ since the automorphisms on $F_{25}$ are id and $\theta :x\to x^{5}$.*

The map $\varphi :R_{q}⟶F_{q}^{2}$ such that $\varphi \left(r_{0}\pi_{0}+r_{1}\pi_{1}\right)=\left(r_{0},r_{1}\right)$ is a ring epimorphism and can be extended to $R_{q}^{n}$ as

$$\begin{array}{ccc} \Phi & : & R_{q}^{n}⟶F_{q}^{2n}\\ \Phi \left(r_{0,0}\pi_{0}+r_{0,1}\pi_{1},\ldots ,r_{n-1,0}\pi_{0}+r_{n-1,1}\pi_{1}\right) & ⟼ & \left(r_{0,0},\ldots ,r_{n-1,0},r_{0,1},\ldots ,r_{n-1,1}\right)=\left(\Phi_{0}|\Phi_{1}\right). \end{array}$$

This Gray type map is an isomorphism of vector spaces over $F_{q}.$ The Gray weight of any element $r\in R_{q}^{n}$ is defined as $w_{G}\left(r\right)=w_{H}\left(\Phi \left(r\right)\right).$ It is apparent that the linear Gray type map Φ is a weight preserving map from $R_{q}^{n}$ to $F_{q}^{2n}.$ A linear code C of length *n* is an $R_{q}$-submodule of $R_{q}^{n}.$ The Euclidean dual of a linear code C over $R_{q}$ of length *n* is defined by $C^{⊥}=\left\{\left.s\in R_{q}^{n}\right|\sum_{i=0}^{n-1}r_{i}s_{i}=0,\forall r\in C\right\}$. Let $\bar{r}=\left({\bar{r}}_{0},{\bar{r}}_{1},\ldots ,{\bar{r}}_{n-1}\right)$ for a vector $r=\left(r_{0},r_{1},\ldots ,r_{n-1}\right)\in R_{q^{2}}^{n}$ where ${\bar{r}}_{i}=r_{i,0}^{q}\pi_{0}+r_{i,1}^{q}\pi_{1}$. The Hermitian dual of a linear code C over $R_{q^{2}}$ of length *n* is defined by $C^{{⊥}_{h}}=\left\{s\in R_{q^{2}}^{n}:\sum_{i=0}^{n-1}r_{i}{\bar{s}}_{i}=0,\;\forall r\in C\right\}$. Note that Euclidean (resp. Hermitian) dual of a linear code over $R_{q}$ (resp. $R_{q^{2}}$) is also linear code over $R_{q}$ (resp. $R_{q^{2}}$).

**Proposition** **1.** *Let C be a linear code of length n over $R_{q}$. Then, $\Phi \left(C^{⊥}\right)=\left(\Phi \left(C\right)\right){}^{⊥}.$ Further, C is a self-dual code iff $\Phi \left(C\right)$ is a self-dual code of length 2n.*

**Proof of Proposition 1.**  It is enough to show that the map Φ preserves the orthogonality, that is, $\langle \Phi \left(c_{0}\right),\Phi \left(c_{1}\right)\rangle =0$ when $\langle c_{0},c_{1}\rangle =0$. By the linearity of Φ, let $r=r_{0}\pi_{0}+r_{1}\pi_{1},s=s_{0}\pi_{0}+s_{1}\pi_{1}\in R_{q}$ such that $\langle r,s\rangle =0$. Then, we get

$$\begin{array}{ccc} \langle r,s\rangle & = & r_{0}s_{0}\pi_{0}+r_{1}s_{1}\pi_{1}\\ & = & \frac{r_{0}s_{0}+r_{1}s_{1}}{2}+\frac{\left(r_{0}s_{0}-r_{1}s_{1}\right)b}{2a}v=0 \end{array}$$

and so $r_{0}s_{0}+r_{1}s_{1}=0$. In this case, it follows that $\langle \Phi \left(r\right),\Phi \left(s\right)\rangle =r_{0}s_{0}+r_{1}s_{1}=0$, which completes the proof. □

Since $R_{q}=\pi_{0}R_{q}⊕\pi_{1}R_{q}$ it follows that $R_{q}^{n}=\pi_{0}R_{q}^{n}⊕\pi_{1}R_{q}^{n}.$ Let C be a linear code of length *n* over $R_{q}$ and $r=\left(r_{0},r_{1},\ldots ,r_{n-1}\right)\in C.$ Then $r_{i}=r_{i,0}\pi_{0}+r_{i,1}\pi_{1},$ where $r_{i,0}$, $r_{i,1}\in F_{q}$ and $r=\left(r_{0,0},r_{1,0},\ldots ,r_{n-1,0}\right)\pi_{0}+\left(r_{0,1},r_{1,1},\ldots ,r_{n-1,1}\right)\pi_{1}.$ Let $C_{i}=\Phi_{i}\left(C\right)$ for i=0,1. It is obvious that $C_{0}$ and $C_{1}$ are linear codes of length *n* over $F_{q}$ and $C=\pi_{0}C_{0}⊕\pi_{1}C_{1}$. This implies that for any linear code C over $R_{q}$ of length *n* there exist linear codes $C_{0}$ and $C_{1}$ over $F_{q}$ of length *n* such that $C=\pi_{0}C_{0}⊕\pi_{1}C_{1}$. The following determines the duals of linear codes over $R_{q}$.

**Proposition** **2.** *Let $C=\pi_{0}C_{0}⊕\pi_{1}C_{1}$ be a linear code of length n over $R_{q}$. Then $C{}^{⊥}=\pi_{0}C_{0}^{{}^{⊥}}⊕\pi_{1}C_{1}^{{}^{⊥}}.$ Further, C is a self-dual code iff both $C_{0}$ and $C_{1}$ are self dual.*

## 4. Skew Constacyclic Codes over the Ring $R_{q}$

In this section, first we will introduce basics of the skew constacyclic codes over finite fields, which are important for determining the algebraic structure of the skew constacyclic codes over non-chain ring $R_{q}.$

For a given automorphism θ of $F_{q},$ the set $F_{q}\left[x;\theta \right]=\left\{a_{0}+a_{1}x+\ldots +a_{1}x^{n}|a_{i}\in F_{q}\;\mathrm{and}\;n\in N\right\}$ of formal polynomials forms a ring with identity under the usual addition of polynomials and the polynomial multiplication with the restriction xb=θ(b)x. The multiplication is extended to all the elements of $F_{q}\left[x;\theta \right]$ via distributivity and associativity. This ring is called the *skew polynomial ring* over $F_{q}.$

**Definition** **1.** For a given element $\lambda \in F_{q}^{*}$ and an automorphism θ of $F_{q}$, a θ-skew λ-constacyclic code over the finite field $F_{q}$ of length n is a linear code C satisfying that $\left(\lambda \theta \left(c_{n-1}\right),\theta \left(c_{0}\right),\ldots ,\theta \left(c_{n-2}\right)\right)\in C$ for each codeword $c=\left(c_{0},\ldots ,c_{n-1}\right)\in C.$

By the definition of a θ-skew λ-constacyclic code C over $F_{q}$, each codeword $c=\left(c_{0},\ldots ,c_{n-1}\right)\in C$ can be considered as a skew polynomial $c\left(x\right)=c_{0}+c_{1}x+\cdots +c_{n-1}x^{n-1}$ in the skew quotient ring $F_{q}\left[x,\theta \right]/\langle x^{n}-\lambda \rangle .$

For the purpose of characterization of skew constacyclic codes over $R_{q},$ we recollect some well known results about skew-constacyclic codes over finite fields [8,13,14,15,47,48,49].

The skew reciprocal polynomial of a polynomial $g\left(x\right)=\sum_{i=0}^{n-k}g_{i}x^{i}\in F_{q}\left[x,\theta \right]$ of degree n−k denoted by $g^{*}\left(x\right)$ is defined as

$$g^{*}\left(x\right)=\sum_{i=0}^{n-k}x^{n-k-i}g_{i}=\sum_{i=0}^{n-k}\theta^{i}\left(g_{n-k-i}\right)x^{i}.$$

If $g_{0}\neq 0$, the left monic skew reciprocal polynomial of g(x) is $g^{♮}\left(x\right):=\frac{1}{\theta^{n-k}\left(g_{0}\right)}g^{*}\left(x\right)$ (see Definition 3 [47]).

From the reference [14], we have the following result.

**Proposition** **3** [14]**.** *Let C be a θ-skew λ-constacyclic code of length n over $F_{q}.$ Then there exists a monic polynomial g(x) of minimal degree in C such that g(x) is a right divisor of $x^{n}-\lambda$ and C=〈g(x)〉.*

Let $g\left(x\right)=x^{m}+g_{m-1}x^{m-1}+\cdots +g_{0}$ be a generator of a θ-skew λ-constacyclic code of length *n* over $F_{q}$. It follows from $x^{n}-\lambda =h\left(x\right)g\left(x\right)$ for some $h\left(x\right)\in F_{q}\left[x,\theta \right]$ that the constant term $g_{0}$ of g(x) can not be zero in $F_{q}$. From [47], we have the following result on the duals of θ-skew λ-constacyclic codes over $F_{q}$.

**Proposition** **4** (Theorem 1 [47])**.** Let C be a θ-skew λ-constacyclic code of length n over $F_{q}$ generated by a monic polynomial g(x) of degree n−k with $g\left(x\right)=x^{n-k}+\sum_{i=0}^{n-k-1}g_{i}x^{i}.$ Let $\lambda^{*}=\frac{\theta^{n}\left(g_{0}\right)}{g_{0}\theta^{n-k}\left(\lambda \right)}.$ Then $C^{⊥}$ is a θ-skew $\lambda^{*}$-constacyclic code of length n over $F_{q}$ such that $C^{⊥}=\langle h^{*}\left(x\right)\rangle$ where h(x) is a monic polynomial of degree k such that $x^{n}-\theta^{-k}\left(\lambda \right)=g\left(x\right)h\left(x\right).$ Moreover $h^{*}\left(x\right)$ is a right divisor of $x^{n}-\lambda^{*}.$

**Definition** **2.** *Let C be a linear code of length 2n over $F_{q},$ and $\lambda_{0},\lambda_{1}$ be units in $F_{q}$ and $\left(\theta ,\sigma \right)\in Aut\left(F_{q}\right)\times S_{2}.$ The code C is called $\left(\lambda_{0},\lambda_{1}\right)$-double twisted with respect to $\left(\theta ,\sigma \right)$ if for all $c=\left(c_{0},c_{1}\right)\in C,$ where $c_{0}=\left(c_{0,0},c_{1,0},\ldots ,c_{n-1,0}\right)$ and $c_{1}=\left(c_{0,1},c_{1,1},\ldots ,c_{n-1,1}\right),$ the word*

$$\left(\begin{array}{c} \lambda_{0}\theta {\left(c\right)}_{n-1,\sigma^{-1}\left(0\right)},\theta {\left(c\right)}_{1,\sigma^{-1}\left(0\right)},\ldots ,\theta {\left(c\right)}_{n-2,\sigma^{-1}\left(0\right)},\\ \lambda_{1}\theta {\left(c\right)}_{n-1,\sigma^{-1}\left(1\right)},\theta {\left(c\right)}_{1,\sigma^{-1}\left(1\right)},\ldots ,\theta {\left(c\right)}_{n-2,\sigma^{-1}\left(1\right)} \end{array}\right)\in C.$$

Now, we give the definition of skew constacyclic codes over $R_{q}$ below.

**Definition** **3.** *Let $\lambda \in U\left(R_{q}\right)$ and $\Theta_{\theta ,\sigma }\in Aut\left(R_{q}\right)$. A linear code C of length n over $R_{q}$ is said to be a $\Theta_{\theta ,\sigma }$-skew λ-constacyclic code of length n over $R_{q}$ if $\left(c_{0},c_{1},\ldots ,c_{n-1}\right)\in C,$ then $\left(\lambda \Theta_{\theta ,\sigma }\left(c_{n-1}\right),\Theta_{\theta ,\sigma }\left(c_{0}\right),\ldots ,\Theta_{\theta ,\sigma }\left(c_{n-2}\right)\right)\in C$.*

We investigate the Φ-Gray images of $\Theta_{\theta ,\sigma }$-skew λ-constacyclic codes over $R_{q}$.

**Proposition** **5.** *Let $\lambda =\lambda_{0}\pi_{0}+\lambda_{1}\pi_{1}\in U\left(R_{q}\right)$ and $\Theta_{\theta ,\sigma }\in Aut\left(R_{q}\right)$. Suppose that $C=\pi_{0}C_{0}⊕\pi_{1}C_{1}$ be a $\Theta_{\theta ,\sigma }$-skew λ-constacyclic code of length n over $R_{q}$. Then*

$$\Phi \left(C\right)=\left\{\Phi \left(c\right):\forall c\in C\right\}$$

*is a*$\left(\lambda_{0},\lambda_{1}\right)$*-double twisted code of length* 2n *over* $F_{q}$ *with respect to* $\left(\theta ,\sigma \right)$.

**Proof of Proposition 5.**  Let $c=\left(c_{0},c_{1}\right)\in \Phi \left(C\right)$ where $c_{i}=\left(c_{0,i},c_{1,i},\ldots ,c_{n-1,i}\right)\in F_{q}^{n}$. Then, $\pi_{0}c_{0}+\pi_{1}c_{1}\in C$. Since C is a $\Theta_{\theta ,\sigma }$-skew λ-constacyclic code over $R_{q}$, we get

$$\begin{array}{ccc} & & \left(\lambda \sum_{i=0}^{1}\pi_{\sigma \left(i\right)}\theta \left(c_{0,i}\right),\sum_{i=0}^{1}\pi_{\sigma \left(i\right)}\theta \left(c_{1,i}\right),\ldots ,\sum_{i=0}^{1}\pi_{\sigma \left(i\right)}\theta \left(c_{n-1,i}\right)\right)\\ & = & \left(\sum_{i=0}^{1}\lambda_{i}\pi_{i}\theta \left(c_{0,\sigma^{-1}\left(i\right)}\right),\sum_{i=0}^{1}\pi_{i}\theta \left(c_{1,\sigma^{-1}\left(i\right)}\right),\ldots ,\sum_{i=0}^{1}\pi_{i}\theta \left(c_{n-1,\sigma^{-1}\left(i\right)}\right)\right)\\ & = & \left(\lambda_{0}\theta \left(c_{0,\sigma^{-1}\left(0\right)}\right),\theta \left(c_{1,\sigma^{-1}\left(0\right)}\right),\ldots ,\theta \left(c_{n-1,\sigma^{-1}\left(0\right)}\right)\right)\pi_{0}+\\ & & \left(\lambda_{1}\theta \left(c_{0,\sigma^{-1}\left(1\right)}\right),\theta \left(c_{1,\sigma^{-1}\left(1\right)}\right),\ldots ,\theta \left(c_{n-1,\sigma^{-1}\left(1\right)}\right)\right)\pi_{1}\in R_{q}. \end{array}$$

Therefore, we have

$$\left(\begin{array}{c} \lambda_{0}\theta {\left(c\right)}_{n-1,\sigma^{-1}\left(0\right)},\theta {\left(c\right)}_{1,\sigma^{-1}\left(0\right)},\ldots ,\theta {\left(c\right)}_{n-2,\sigma^{-1}\left(0\right)},\\ \lambda_{1}\theta {\left(c\right)}_{n-1,\sigma^{-1}\left(1\right)},\theta {\left(c\right)}_{1,\sigma^{-1}\left(1\right)},\ldots ,\theta {\left(c\right)}_{n-2,\sigma^{-1}\left(1\right)} \end{array}\right)\in \Phi \left(C\right),$$

which completes the proof. □

As an immediate result of Proposition 5, letting σ=id and $\Theta_{\theta ,id}=\Theta_{\theta }$ we deduce the following theorem:

**Theorem** **2.** *Let $\lambda =\lambda_{0}\pi_{0}+\lambda_{1}\pi_{1}\in U\left(R_{q}\right)$ and $\Theta_{\theta }\in Aut\left(R_{q}\right)$. Suppose that $C=\pi_{0}C_{0}⊕\pi_{1}C_{1}$ be a linear code of length n over $R_{q}$. Then, C is a $\Theta_{\theta }$-skew λ-constacyclic code over $R_{q}$ of length n if and only if $C_{i}$ is a θ-skew $\lambda_{i}$-constacyclic code over $F_{q}$ of length n.*

**Proof of Theorem 2.**  It follows from the proof of Proposition 5 by taking σ=id. □

Hereafter, we only consider the automorphism $\Theta_{\theta }=\Theta_{\theta ,id}$ defined by

$$\begin{array}{ccc} \Theta_{\theta } & : & R_{q}⟶R_{q}\\ \Theta_{\theta }\left(r_{0}\pi_{0}+r_{1}\pi_{1}\right) & \mapsto & \theta \left(r_{0}\right)\pi_{0}+\theta \left(r_{1}\right)\pi_{1}, \end{array}$$

where $\theta \in Aut\left(F_{q}\right)$.

Now, we give a generator of a $\Theta_{\theta }$-skew λ-constacyclic code over $R_{q}$, where $\lambda =\lambda_{0}\pi_{0}+\lambda_{1}\pi_{1}\in U\left(R_{q}\right)$.

**Proposition** **6.** *Let $\lambda =\lambda_{0}\pi_{0}+\lambda_{1}\pi_{1}\in U\left(R_{q}\right)$ and $\Theta_{\theta }\in Aut\left(R_{q}\right)$. Suppose that $C=\pi_{0}C_{0}⊕\pi_{1}C_{1}$ be a $\Theta_{\theta }$-skew λ-constacyclic code of length n over $R_{q}$. Then there exist polynomials $g_{0}\left(x\right)$ and $g_{1}\left(x\right)\in F_{q}\left[x,\theta \right]$ such that $C=\langle \pi_{0}g_{0}\left(x\right),\pi_{1}g_{1}\left(x\right)\rangle$ with $C_{i}=\langle g_{i}\rangle \subseteq F_{q}\left[x,\theta \right]/\langle x^{n}-\lambda_{i}\rangle$.*

**Proof of Proposition 6.**  Let $E=\langle \pi_{0}g_{0}\left(x\right),\pi_{1}g_{1}\left(x\right)\rangle$ and let $c\left(x\right)=\pi_{0}c_{0}\left(x\right)+\pi_{1}c_{1}\left(x\right)\in C$ such that $c_{i}\left(x\right)\in C_{i}.$ Since $C_{i}=\langle g_{i}\rangle$ is a left submodule of the skew ring $F_{q}\left[x,\theta \right]/\langle x^{n}-\lambda_{i}\rangle ,$ there exist $l_{0}$ and $l_{1}\in F_{q}\left[x,\theta \right]$ such that $c\left(x\right)=l_{0}\left(x\right)\pi_{0}c_{0}\left(x\right)+l_{1}\left(x\right)\pi_{1}c_{1}\left(x\right)\in E$ and hence C⊂E.

On the other hand, let e∈E, then there exist $k_{0}$ and $k_{1}\in F_{q}\left[x,\theta \right]/\langle x^{n}-\lambda_{i}\rangle$ such that $e\left(x\right)=k_{0}\left(x\right)g_{0}\left(x\right)\pi_{0}+$$k_{1}\left(x\right)g_{1}\left(x\right)\pi_{1}$. Then there exist $b_{0}$ and $b_{1}\in F_{q}\left[x,\theta \right]$ such that $\pi_{i}b_{i}\left(x\right)=\pi_{i}k_{i}\left(x\right)$, thus

$$e\left(x\right)=b_{0}\left(x\right)g_{0}\left(x\right)\pi_{0}+b_{1}\left(x\right)g_{1}\left(x\right)\pi_{1}\in C.$$

This shows that E⊂C. □

We give the exact characterization of $\Theta_{\theta }$-skew λ-constacyclic codes over $R_{q}$ as a consequence of Proposition 6.

**Theorem** **3.** *Let $\lambda =\lambda_{0}\pi_{0}+\lambda_{1}\pi_{1}\in U\left(R_{q}\right)$ and $\Theta_{\theta }\in Aut\left(R_{q}\right)$. Suppose that $C=\pi_{0}C_{0}⊕\pi_{1}C_{1}$ be a $\Theta_{\theta }$-skew λ-constacyclic code of length n over $R_{q}$. Then C is principally generated with $C=\langle g\left(x\right)\rangle$, where $g\left(x\right)=\pi_{0}g_{0}\left(x\right)+\pi_{1}g_{1}\left(x\right)$ and $g\left(x\right)$ is a right divisor of $x^{n}-\lambda$ in $R_{q}\left[x,\Theta_{\theta }\right]$.*

**Proof of Theorem 3**  It is apparent that $\langle g\left(x\right)\rangle \subset C$. Since $\pi_{i}g\left(x\right)=\pi_{i}g_{i}\left(x\right)$ for i=0,1, we have $C\subset \langle g\left(x\right)\rangle .$ This implies that $C=\langle g\left(x\right)\rangle .$ Since $g_{i}\left(x\right)$ is a right divisor of $x^{n}-\lambda_{i},$ there exists $h_{i}\left(x\right)\in F_{q}\left[x,\theta \right]$ such that $x^{n}-\lambda_{i}=h_{i}\left(x\right)g_{i}\left(x\right).$ Seeing that $\pi_{i}\left(x^{n}-\lambda \right)=\pi_{i}\left(x^{n}-\lambda_{i}\right)$, hence

$$\begin{array}{ccc} \left(\pi_{0}h_{0}\left(x\right)+\pi_{1}h_{1}\left(x\right)\right)\left(\pi_{0}g_{0}\left(x\right)+\pi_{1}g_{1}\left(x\right)\right) & = & \pi_{0}h_{0}\left(x\right)g_{0}\left(x\right)+\pi_{1}h_{1}\left(x\right)g_{1}\left(x\right)\\ & = & \pi_{0}\left(x^{n}-\lambda_{0}\right)+\pi_{1}\left(x^{n}-\lambda_{1}\right)\\ & = & \pi_{0}\left(x^{n}-\lambda \right)+\pi_{1}\left(x^{n}-\lambda \right)\\ & = & \left(\pi_{0}+\pi_{1}\right)\left(x^{n}-\lambda \right)\\ & = & x^{n}-\lambda . \end{array}$$

This shows that $\pi_{0}h_{0}\left(x\right)+\pi_{1}h_{1}\left(x\right)$ is a right divisor of $x^{n}-\lambda .$ □

Proposition 2, Proposition 3, Theorem 2 and Theorem 3 together imply the following result:

**Theorem** **4.** Let $\lambda =\lambda_{0}\pi_{0}+\lambda_{1}\pi_{1}\in U\left(R_{q}\right)$ and $\Theta_{\theta }\in Aut\left(R_{q}\right)$. If $C=\pi_{0}C_{0}⊕\pi_{1}C_{1}$ is a $\Theta_{\theta }$-skew λ-constacyclic code of length n over $R_{q}$ with $C_{i}=\langle g_{i}\left(x\right)\rangle$, $g_{i}\left(x\right)=x^{n-k_{i}}+\sum_{j=0}^{n-k_{i}-1}g_{ij}x^{j}$, then $C^{⊥}=\langle h^{*}\left(x\right)\rangle$ is a $\Theta_{\theta }$-skew $\lambda^{*}$-constacyclic code of length n over $R_{q}$, where $\lambda^{*}=\sum_{i=0}^{1}\frac{\theta^{n}\left(g_{i0}\right)}{g_{i0}\theta^{n-k_{i}}\left(\lambda_{i}\right)}\pi_{i}$ and $h^{*}\left(x\right)=\sum_{i=0}^{1}\pi_{i}h_{i}^{*}\left(x\right)$.

**Proof of Theorem 4**  Recall that $C^{⊥}=\pi_{0}C_{0}^{⊥}+\pi_{1}C_{1}^{⊥}$ by Proposition 2 and $C_{i}=\langle h_{i}^{*}\rangle$ is a $\Theta_{\theta }$-skew λ-constacyclic code over $F_{q}$ by Proposition 4. Then, by Theorem 2, $C^{⊥}$ is a $\Theta_{\theta }$-skew $\lambda^{*}$-constacyclic code over $R_{q}$. Finally, Theorem 3 gives the generator polynomial $h^{*}\left(x\right)$ of $C^{⊥}$. □

## 5. Linear Complementary Dual Skew Constacyclic Codes over $R_{q}$

In this section, we define LCD codes over $R_{q}$ and give a characterization for skew constacyclic codes over $R_{q}$ to be Euclidean LCD and Hermitian LCD. Before giving the definition of LCD codes over $R_{q}$, we briefly state some basic definitions and results on LCD codes over $F_{q}$.

A linear code C over $F_{q}$ is said to be an Euclidean LCD code if the intersection of C and $C^{⊥}$ is zero, that is, $Hull\left(C\right)=C\cap C^{⊥}=\left\{0\right\}$ [16]. A Hermitian LCD code is a linear code *C* over $F_{q^{2}}$ with $Hull_{h}\left(C\right)=C\cap C^{{⊥}_{h}}=\left\{0\right\}$. From [50], we have the following theorem for skew constacyclic codes over finite fields to be Euclidean LCD and Hermitian LCD.

**Theorem** **5** (Theorem 2 [50])**.** *Let* $\theta \in Aut\left(F_{q}\right)$ *and* $\lambda \in \left\{\;\pm 1\right\}$. *Let* C *be a θ-skew λ-constacyclic code of length n over* $F_{q}$ *with* $C=\langle g\left(x\right)\rangle$. *Let* $h\left(x\right)\in F_{q}\left[x;\theta \right]$ *with* $\theta^{n}\left(h\left(x\right)\right)g\left(x\right)=x^{n}-\lambda$. *Then,*

(1) *C is an Euclidean LCD code ⇔$gcrd\left(g,h^{♮}\right)=1$. (Here, $gcrd(g,h^{♮})$ represents the greatest common right divisor of g and $h^{♮}.$)*
(2) *Let q be an even power of a prime number. Then, C is a Hermitian LCD code ⇔$gcrd\left(g,{\tilde{h}}^{♮}\right)=1$. (For $a\left(x\right)=\sum a_{i}x^{i}$, $\tilde{a}\left(x\right)=\sum a_{i}^{q}x^{i}$.)*

The definitions of Euclidean LCD and Hermitian LCD codes over $R_{q}$ are similar to the ones over finite fields.

**Definition** **4.** *A linear code C over $R_{q}$ (resp. $R_{q^{2}}$) is called an Euclidean (resp. Hermitian) LCD code if $C\cap C^{⊥}=\left\{0\right\}$ (resp. $C\cap C^{{⊥}_{h}}=\left\{0\right\}$).*

The following explores when a linear code over $R_{q}$ is an Euclidean LCD or a Hermitian LCD.

**Proposition** **7.** *Let $C=\pi_{0}C_{0}⊕\pi_{1}C_{1}$ be a linear code over $R_{q}$ (resp. $R_{q^{2}}$). Then, C is an Euclidean (resp. Hermitian) LCD code over $R_{q}$ (resp. $R_{q^{2}}$) if and only if $C_{i}$’s are Euclidean (resp. Hermitian) LCD codes over $F_{q}$ (resp. $F_{q^{2}}$).*

**Proof of Proposition 7.**  Since $C^{⊥}=\pi_{0}C_{0}^{⊥}⊕\pi_{1}C_{1}^{⊥}$ by Proposition 2, we get

$$\begin{array}{ccc} C\cap C^{⊥} & = & \left(\pi_{0}C_{0}⊕\pi_{1}C_{1}\right)\cap \left(\pi_{0}C_{0}^{⊥}⊕\pi_{1}C_{1}^{⊥}\right)\\ & = & \left(C_{0}\cap C_{0}^{⊥}\right)\pi_{0}⊕\left(C_{1}\cap C_{1}^{⊥}\right)\pi_{1}, \end{array}$$

which implies that $C\cap C^{⊥}=\left\{0\right\}$⇔$C_{i}\cap C_{i}^{⊥}=\left\{0\right\}$. The Hermitian case is similar. □

**Theorem** **6.** *Let $\lambda =\lambda_{0}\pi_{0}+\lambda_{1}\pi_{1}\in \left\{\mp 1,\mp \frac{b}{a}v\right\}\subset U\left(R_{q}\right)$ and $\Theta_{\theta }\in Aut\left(R_{q}\right)$. A $\Theta_{\theta }$-skew λ-constacyclic code $C=\pi_{0}C_{0}⊕\pi_{1}C_{1}$ of length n over $R_{q}$ (resp. over $R_{q^{2}}$), where $C_{i}=\langle g_{i}\left(x\right)\rangle$ and $\theta^{n}\left(h_{i}\left(x\right)\right)g_{i}\left(x\right)=x^{n}-\lambda_{i}$, is an Euclidean (resp. Hermitian) LCD code over $R_{q}$ if and only if $gcrd\left(g_{i}\left(x\right),h_{i}^{♮}\left(x\right)\right)=1$ (resp. $gcrd\left(g_{i}\left(x\right),{\tilde{h}}_{i}^{♮}\left(x\right)\right)=1$).*

**Proof of Theorem 6.**  See that $\lambda_{i}\in \left\{\mp 1\right\}$ if $\lambda =\lambda_{0}\pi_{0}+\lambda_{1}\pi_{1}\in \left\{\mp 1,\mp \frac{b}{a}v\right\}\subset U\left(R_{q}\right)$. The remain of the proof follows from Proposition 7 and Theorem 5. The Hermitian case is similar. □

We also have the following result from Proposition 7.

**Theorem** **7.** *Let $C=\pi_{0}C_{0}⊕\pi_{1}C_{1}$ be a linear code over $R_{q}$ (resp. $R_{q^{2}}$). Then, C is an Euclidean (resp. Hermitian) LCD code over $R_{q}$ (resp. $R_{q^{2}}$) if and only if $\Phi \left(C\right)$ is an Euclidean (resp. Hermitian) LCD codes over $F_{q}$ (resp. $F_{q^{2}}$).*

**Example** **2.** *Let a=3, b=2, p=13 and q=169. Then, $\pi_{0}=7+9v$ and $\pi_{1}=7+4v$. Let $F_{169}=\left\{x+yw\;|\;x,y\in F_{13}\right\}$, where $w^{2}-w+2=0$. Let $\theta :x\to x^{13}\in Aut\left(F_{169}\right)$ be Frobenius automorphism. Observe that $\left(x^{3}+w^{150}x^{2}+12x+w^{66}\right)\left(x+w^{18}\right)=x^{4}-1$ and $\left(x^{3}+w^{33}x^{2}+5x+w^{159}\right)\left(x+w^{9}\right)=x^{4}+1$ in $F_{169}\left[x,\theta \right]$. Let $C_{0}=\langle g_{0}\left(x\right)=x+w^{18}\rangle$ and $C_{1}=\langle g_{1}\left(x\right)=x+w^{9}\rangle$ be an Euclidean LCD θ-skew cyclic code and an Euclidean LCD θ-skew negacyclic (λ=−1) code of length 4 over $F_{169}$, respectively. Then $C=\pi_{0}C_{0}⊕\pi_{1}C_{1}$ is an Euclidean LCD $\Theta_{\theta }$-skew 5v-constacyclic code of length 4 over $R_{169}$ with generator polynomial $g\left(x\right)=x+w^{139}+w^{152}v$ and $\Phi \left(C\right)$ is an Euclidean LCD code with parameters ${\left[8,6,2\right]}_{169}$, which is almost MDS. Moreover, we list some Euclidean LCD $\Theta_{\theta }$-skew constacyclic codes over $R_{169}$ of length 4 and present the parameters of almost MDS and near MDS Euclidean LCD codes over $F_{169}$ of length 8 obtained via the map Φ in Table 1.*

## 6. Entanglement-Assisted Quantum Codes with Maximal Entanglement from Skew Constacyclic LCD Codes over $R_{q}$

In this section, we apply the skew constacyclic LCD codes over $R_{q}$ to obtain parameters for the entanglement assisted quantum codes with maximal entanglement [28].

An ${\left[\;\left[n,k,d;c\right]\;\right]}_{q}$ EAQECC is a quantum code that encodes *k* information qubits into *n* qubits and corrects up to $\left\lfloor \frac{d-1}{2}\right\rfloor$ errors via *c* pairs of maximally entanglement states. For an ${\left[\;\left[n,k,d;c\right]\;\right]}_{q}$ EAQECC, the number *c* of maximally entanglement states based on the linear codes is less than or equal to n−k, and if c=n−k, then this is called an EAQECC with maximal entanglement [51]. We have the following construction for EAQECCs obtained from linear codes over $F_{q^{2}}$.

**Theorem** **8** [45]**.** *If there exists an ${\left[n,k,d\right]}_{q^{2}}$ linear code with parity check matrix H, then there exists an EAQECC having parameters ${\left[\;\left[n,2k-n+c,d;c\right]\;\right]}_{q}$, where $c=rank\left(HH^{†}\right)$.*

We also have the following from [Proposition 3.2] [34].

**Proposition** **8** [34]**.** *If C is a ${\left[n,k,d\right]}_{q^{2}}$ linear code with parity check matrix H, then $rank\left(HH^{†}\right)=n-k-\dim\left(Hull_{h}\left(C\right)\right)$.*

Theorem 8 and Proposition 8 together imply that since $\dim\left(Hull_{h}\left(C\right)\right)=0$ and so $c=rank\left(HH^{†}\right)=n-k$ for an ${\left[n,k,d\right]}_{q^{2}}$ Hermitian LCD code, one gets an ${\left[\;\left[n,k,d;n-k\right]\;\right]}_{q}$ EAQECC. Since the Φ-images of the Hermitian LCD codes over $R_{q^{2}}$ are also Hermitian LCD codes over $F_{q^{2}}$, we derive a family of EAQECCs from $\Theta_{\theta }$-skew λ-constacyclic codes over $R_{q^{2}}$ as following.

**Theorem** **9.** *Let $\lambda =\lambda_{0}\pi_{0}+\lambda_{1}\pi_{1}\in \left\{\mp 1,\mp \frac{b}{a}v\right\}\subset U\left(R_{q}\right)$ and $\Theta_{\theta }\in Aut\left(R_{q}\right)$. Let $C=\pi_{0}C_{0}⊕\pi_{1}C_{1}$ be a $\Theta_{\theta }$-skew λ-constacyclic code of length n over $R_{q^{2}}$, where $C_{i}=\langle g_{i}\left(x\right)\rangle$ and $\theta^{n}\left(h_{i}\left(x\right)\right)g_{i}\left(x\right)=x^{n}-\lambda_{i}$. If $gcrd\left(g_{i}\left(x\right),{\tilde{h}}_{i}^{♮}\left(x\right)\right)=1$, then there exists a maximal entanglement EAQECC having parameters ${\left[\;\left[2n,k_{0}+k_{1},d;2n-k_{0}-k_{1}\right]\;\right]}_{q}$, where $\deg\left(g_{i}\left(x\right)\right)=n-k_{i}$, $d=\min\left\{d_{0},d_{1}\right\}$ and $d_{i}$ is the minimum distance of $C_{i}$.*

**Example** **3.** *Let a=2, b=1, p=5 and q=25. Then, $\pi_{0}=3+4v$ and $\pi_{1}=3+v$. Let $F_{25}=\left\{x+yw\;|\;x,y\in F_{5}\right\}$, where $w^{2}-w+2=0$. Let $\theta :x\to x^{5}\in Aut\left(F_{25}\right)$ be Frobenius automorphism. See that $\left(x^{3}+w^{17}x^{2}+x+4\right)\left(x^{3}+wx^{2}+x+1\right)=x^{6}-1$ and $\left(x^{4}+w^{9}x^{3}+w^{23}x^{2}+w^{9}x+w^{4}\right)\left(x^{2}+w^{21}x+w^{2}0\right)=x^{6}+1$ in $F_{25}\left[x,\theta \right]$. Note that $C_{0}=\langle g_{0}\left(x\right)=x^{3}+wx^{2}+x+1\rangle$ and $C_{1}=\langle g_{1}\left(x\right)=x^{2}+w^{21}x+w^{20}\rangle$ be a Hermitian LCD θ-skew cyclic code and a Hermitian LCD θ-skew negacyclic (λ=−1) code of length 6 over $F_{25}$, respectively. Then $C=\pi_{0}C_{0}⊕\pi_{1}C_{1}$ is a Hermitian LCD $\Theta_{\theta }$-skew 3v-constacyclic code of length 6 over $R_{25}$ with generator polynomial $g\left(x\right)=\left(3+4v\right)x^{3}+\left(w^{16}+w^{5}v\right)x^{2}+\left(w+w^{23}v\right)x+\left(w^{13}+w^{16}v\right)$ and $\Phi \left(C\right)$ is a Hermitian LCD code with parameters ${\left[12,7,3\right]}_{25}$. Applying Theorem 9, we get an ${\left[\;\left[12,7,3;5\right]\;\right]}_{5}$ EAQECC with maximal entanglement. Furthermore, we list some Hermitian LCD $\Theta_{\theta }$-skew constacyclic codes over $R_{25}$ of length 6 and present the parameters of EAQECCs with maximal entanglement over $F_{5}$ of length 12 obtained via the map Φ and Theorem 9 in Table 2.*

## 7. Conclusions

In this paper, by determining the automorphism group of the ring $R_{q}$ we define and study the skew constacyclic codes over $R_{q}$. We characterize the algebraic structure of skew constacyclic codes and their duals over $R_{q}$. We investigate the Φ-images of skew constacyclic codes over $R_{q}$. Moreover, we consider LCD codes over $R_{q}$ and give a necessary and sufficient condition for skew constacyclic codes $R_{q}$ to be Euclidean and Hermitian LCD. We also give some examples of Euclidean LCD codes over $R_{169}$ of length 4 and tabulate the parameters of almost MDS and near MDS Euclidean LCD codes over $F_{169}$ of length 8 as the Φ-images of these codes over $R_{169}$. Finally, as an application of these Hermitian LCD skew constacyclic codes over $R_{q}$, we obtain a class of EAQECCs with maximal entanglement and tabulate parameters of some EAQECCs with maximal entanglement over $F_{5}$ of length 12. In the process of preparing this study, the following two questions were among those that we could not answer yet, but which offer potential avenues for future research.

(**Q1**) We just determine the algebraic structure of the $\Theta_{\theta ,id}$-skew λ-constacyclic codes over $R_{q}$. What about the algebraic structure of the more general case $\Theta_{\theta ,\sigma }$-skew λ-constacyclic codes over $R_{q}$?
(**Q2**) What about the self-duality of $\Theta_{\theta ,\sigma }$-skew λ-constacyclic codes over $R_{q}$? In this case, does there exists any restriction on λ?

## Figures and Tables

**Table 1:** Generator polynomials of some Euclidean LCD $\Theta_{\theta }$-skew λ-constacyclic codes over $R_{169}$ of length 4 and Euclidean LCD codes over $F_{169}$ of length 8 as their Φ-images. The parameters with “*” and “**” are almost MDS and near MDS, respectively.

$C_{0}=\langle g_{0}\left(x\right)\rangle$	$C_{1}=\langle g_{1}\left(x\right)\rangle$	$C=\langle \pi_{0}g_{0}\left(x\right)+\pi_{1}g_{1}\left(x\right)\rangle$	λ	$\Phi \left(C\right)$
$x+w^{18}$	$x+w^{9}$	$x+w^{139}+w^{152}v$	5v	${\left[8,6,2\right]}_{169}^{*}$
$x^{2}+w^{8}x+w^{6}$	$x^{2}+w^{21}x+w^{12}$	$x^{2}+\left(w^{80}+w^{121}v\right)x+w^{67}+w^{92}v$	5v	${\left[8,4,3\right]}_{169}^{**}$
$x+w^{12}$	$x+w^{3}$	$x+w^{133}+w^{146}v$	5v	${\left[8,6,2\right]}_{169}^{*}$
$x^{2}+w^{9}x+w^{12}$	$x^{2}+wx+w^{18}$	$x^{2}+\left(w^{128}+w^{150}v\right)x+5+w^{30}v$	8v	${\left[8,4,3\right]}_{169}^{**}$
$x^{2}+w^{69}x+w^{12}$	$x^{2}+w^{145}x+w^{18}$	$x^{2}+\left(12+w^{146}v\right)x+5+5+w^{30}v$	8v	${\left[8,4,3\right]}_{169}^{**}$
$x+w^{9}$	$x+w^{24}$	$x+11+w^{72}v$	8v	${\left[8,6,2\right]}_{169}^{*}$
$x+w^{30}$	$x^{2}+x+w^{30}$	$\left(7+4v\right)x^{2}+x+w^{30}$	1	${\left[8,5,2\right]}_{169}^{**}$
$x^{2}+w^{12}x+w^{30}$	$x^{2}+w^{24}x+w^{30}$	$x^{2}+\left(w^{165}+w^{158}v\right)x+w^{30}$	1	${\left[8,4,3\right]}_{169}^{**}$
$x+w^{36}$	x+8	$x+w^{150}+w^{54}v$	1	${\left[8,6,2\right]}_{169}^{*}$
$x+w^{15}$	$x+w^{21}$	$x+w^{129}+w^{33}v$	−1	${\left[8,6,2\right]}_{169}^{*}$
$x^{2}+w^{57}x+w^{156}$	$x^{2}+w^{141}x+w^{12}$	$x^{2}+\left(9+w^{15}v\right)x+9+w^{133}v$	−1	${\left[8,4,3\right]}_{169}^{**}$
$x^{2}+7x+w^{24}$	$x+w^{33}$	$\left(7+9v\right)x^{2}+\left(4+2v\right)x+7+w^{83}v$	−1	${\left[8,5,2\right]}_{169}^{**}$

**Table 2:** Generator polynomials of some Hermitian LCD $\Theta_{\theta }$-skew λ-constacyclic codes over $R_{25}$ of length 6 and some EAQECCs with maximal entanglement over $F_{5}$ of length 12 obtained by Theorem 9.

$C_{0}=\langle g_{0}\left(x\right)\rangle$	$C_{1}=\langle g_{1}\left(x\right)\rangle$	$C=\langle \pi_{0}g_{0}\left(x\right)+\pi_{1}g_{1}\left(x\right)\rangle$	λ	EAQECC
$x^{3}+wx^{2}+x+1$	$x^{2}+w^{21}x+w^{20}$	$\left(3+4v\right)x^{3}+\left(w^{16}+w^{5}v\right)x^{2}+\left(w+w^{23}v\right)x+w^{13}+w^{16}v$	3v	${\left[\;\left[12,7,3;5\right]\;\right]}_{5}$
$x^{2}+w^{3}x+w^{8}$	$x^{2}+w^{17}x+w^{20}$	$x^{2}+\left(1+w^{8}v\right)x+w^{2}$	3v	${\left[\;\left[12,8,3;4\right]\;\right]}_{5}$
$x^{4}+w^{11}x^{3}+w^{11}x^{2}+w^{23}x+w^{4}$	$x^{4}+w^{21}x^{3}+w^{19}x^{2}+w^{21}x+w^{20}$	$x^{4}+\left(3+w^{14}v\right)x^{3}+\left(w^{9}+3v\right)x^{2}+\left(w^{8}+v\right)x+3+w^{15}v$	3v	${\left[\;\left[12,4,5;8\right]\;\right]}_{5}$
$x+w^{4}$	$x+w^{16}$	$x+w^{22}v$	1	${\left[\;\left[12,10,2;2\right]\;\right]}_{5}$
$x^{3}+w^{5}x^{2}+w^{2}x+w^{16}$	$x^{3}+w^{16}x^{2}+wx+w^{16}$	$x^{3}+\left(w^{15}+w^{14}v\right)x^{2}+\left(w^{17}+2v\right)x+w^{16}$	1	${\left[\;\left[12,6,4;6\right]\;\right]}_{5}$
$x^{2}+w^{7}x+w^{16}$	$x^{2}+w^{3}x+w^{16}$	$x^{2}+\left(w^{20}+w^{23}v\right)x+w^{16}$	1	${\left[\;\left[12,8,3;4\right]\;\right]}_{5}$
$x^{2}+w^{21}x+w^{4}$	$x^{2}+w^{17}x+w^{4}$	$x^{2}+\left(w^{10}+w^{13}v\right)x+w^{4}$	−1	${\left[\;\left[12,8,3;4\right]\;\right]}_{5}$
$x^{2}+w^{21}x+w^{4}$	$x^{4}+w^{21}x^{3}+w^{19}x^{2}+w^{21}x+w^{20}$	$\left(3+v\right)x^{4}+\left(w^{15+w^{21}v}\right)x^{3}+\left(w^{3}+w^{20}v\right)x^{2}+w^{21}x+3+w^{15}v$	−1	${\left[\;\left[12,6,4;6\right]\;\right]}_{5}$

## Data Availability

Not applicable.

## Abbreviations

The following abbreviations are used in this manuscript:

LDC	Linear Complementary Dual
EAQECC	Entanglement-Assisted Quantum Error Correcting Code
MDS	Maximum Distance Separable
